# Addition of high-intensity interval training to a moderate intensity continuous training cardiovascular rehabilitation program after ischemic cerebrovascular disease: A randomized controlled trial

**DOI:** 10.3389/fneur.2022.963950

**Published:** 2023-01-04

**Authors:** Thalia Lapointe, Julie Houle, Ying-Tung Sia, Marika Payette, François Trudeau

**Affiliations:** ^1^Department of Human Kinetics, Université du Québec à Trois-Rivières, Trois-Rivières, QC, Canada; ^2^Department of Nursing, Université du Québec à Trois-Rivières, Trois-Rivières, QC, Canada; ^3^Integrated University Center for Health and Social Services Mauricie- and Centre-du-Québec, Trois-Rivières, QC, Canada

**Keywords:** HIIT, stroke, aerobic exercise, deconditioning, cerebrovascular disease

## Abstract

**Introduction:**

Moderate intensity continuous training (MICT) is usually recommended for stroke or transient ischemic attack (TIA) patients. High intensity interval training (HIIT) has emerged as a potentially effective method for increasing cardiorespiratory fitness (CRF) among clinical populations. Its effectiveness remains to be demonstrated after stroke. A combined program of HIIT and MICT was designed to create a realistic exercise program implemented for a clinical setting to help patients become more active.

**Purpose:**

This study aimed to compare the effects of a 6-month exercise program with either MICT only or a combination of HIIT and MICT and a control group in terms of CRF, cardiovascular risk factors, functionality, cognitive function (Montreal Cognitive Assessment) and depression markers (Hospital Anxiety and Depression Scale).

**Methods:**

This randomized controlled trial started with 52 participants (33 men and 19 women, mean age: 69.2 ± 10.7) divided into three groups: HIIT + MICT combined, MICT, and control. Both exercise groups consisted of 4 weekly sessions including supervised and at-home exercise. Outcomes were assessed at T0 (baseline measure), T6 (end of exercise protocols), and T12 (follow-up), 40 participants having completed the 12-month follow-up.

**Results:**

At T6, both HIIT+MICT and MICT programs provided a similar increase of CRF (3 ml·min-1·kg-1) from baseline (*p* < 0.01), while the control group showed a global slight decrease. Despite some decrease of CRF at T12 compared to T6, improvement persisted 6 months post-intervention (HIIT + MICT: *p* < 0.01 and MICT: *p* < 0.05). The control group decreased compared with baseline (*p* < 0.05). The two exercise programs induced a comparable increase in self-reported physical activity and a decrease in anxiety and depression markers. Participants in HIIT + MICT and MICT programs declared a good degree of acceptability assessed by the Acceptability and Preferences Questionnaire.

**Conclusion:**

A 6-month HIIT + MICT combined program and a standard MICT program induced similar improvements in CRF, self-reported physical activity and anxiety and depression markers among patients with prior ischemic stroke or TIA compared with a control group. These effects appear to persist over time. Addition of HIIT was safe and considered acceptable by participants. Our results do not support any superiority of the combination HIIT + MICT nor disadvantage vs. MICT in this population.

## Introduction

Stroke is the second leading cause of death and the first leading cause of disability in the world (1). Annually, 15 million people worldwide experience stroke, and two-thirds of these report important disabilities leading to significant functional deficits (2). Stroke survivors live longer than before, and the World Stroke Organization emphasizes the urgent need to design and implement interventions to improve quality of life after stroke (3). The literature suggests that stroke patients are highly deconditioned and have a reduced cardiorespiratory fitness (CRF), which is around 50% of predicted VO_2_peak values in age-matched sedentary controls in their mid-60s (13.6 ml·min^−1^·kg^−1^ vs. between 25 and 30 ml·min^−1^·kg^−1^) (4, 5). Compared to normative data provided by American College of Sport Medicine (ACSM), Billinger et al. demonstrated that for each decade, stroke survivors have CRF below the first percentile which classified them in the very poor category (6). Such deconditioning limits daily activities, increases sedentary time, and decreases functional autonomy, leading to a vicious cycle that increases cardiovascular risk factors and associated comorbidities and frailty (7). Furthermore, classical cardiovascular risk factors such as hypertension, obesity, dyslipidemia, and impaired fasting blood glucose are highly prevalent in stroke patients, increasing their odds for recurrent strokes or cardiovascular events (8).

The literature indicates that exercise programs are effective in secondary prevention for improving CRF and cardiovascular risk factors following a stroke or transient ischemic attack (TIA) (9). A meta-epidemiological study revealed that exercise may be more effective than drug treatment alone (anticoagulants and antiplatelets) for reducing post-stroke mortality (10). Although it is recognized that aerobic training is a key component of stroke rehabilitation, it continues to be used sporadically in clinical settings, and recommendations about specific intensities are based on moderate level of evidence (11). Low to moderate intensity training is generally recommended in the chronic phase of stroke recovery (40–70% VO_2_ reserve; 55–80% maximal heart rate; rating of perceived exertion 11–14/20) (12). Although high intensity interval training (HIIT) has emerged as a potentially effective and safe alternative that may offer greater improvement in health and CRF in the clinical population (13), the superiority of this training method compared with other exercise modalities and the optimal parameters remain unknown in the population with stroke. A recent randomized control trial showed that 8 weeks of treadmill HIIT was superior to standard care (no exercise program) to increase CRF immediately after the intervention, but the difference between groups was not maintained at a 12-month follow-up (14). Crozier et al. (15) report that HIIT can improve cardiovascular health post-stroke and suggest frequency, intensity, time, and type parameters, but emphasize that individualized protocols are warranted. Although its feasibility and preliminary safety have been studied (16–18), the benefits of combining HIIT and MICT compared with those of MICT alone and its acceptability in stroke patients remain to be established. Acceptability refers to a favorable or positive attitude toward treatment options (19). Treatment preferences are of clinical importance and are an essential part of patient-centered care that contributes to adherence and consequently outcome achievement (20).

An additional concern regarding stroke patients is that psychological depression affects 25–79% of survivors (21), and this condition has a negative effect on functional recovery. Anxiety disorders are also more prevalent in these patients than in the general population (22). Moreover, approximately two-thirds of stroke survivors have a cognitive impairment (23). The literature indicates that physical activity is associated with lower levels of both depression and anxiety among the elderly (24) and can improve cognitive performance in older adults with cognitive impairment (25). However, these associations do not seem clear as regards stroke patients, and the optimal exercise prescription has yet to be determined (26). HIIT has been suggested as a potential beneficial addition for promoting neuroplasticity post-stroke (15), but results are limited to animal models, single-session exercise, or healthy populations. Higher neurotrophins liberation associated with higher exercise intensities, like BDNF, could also influence positively cognitive functions (51).

Some authors recommend caution and professional supervision of post-stroke patients performing high intensity exercise (11) or HIIT (54), while interventional studies failed to find more incidents involving post-stroke patients performing HIIT compared with control groups (55). As moderate intensity continuous training (MICT) is easily transferable to the home, we combined supervised HIIT and unsupervised MICT. This could provide the physiological benefits of HIIT in a clinical setting while allowing patients to continue their daily MICT routine from the comfort of home.

This study aimed to compare the effect of a 6-month exercise program regarding three conditions: a standard program involving MICT, a combination of HIIT and MICT (HIIT + MICT) and a control group. All groups were constituted of patients with prior ischemic stroke or TIA. The principal outcome was CRF measured at baseline (T0), at the end of the exercise program (T6) and 6 months later (T12). Additional outcomes were other modifiable cardiovascular risk factors, self-reported physical activity, physical functionality, anxiety and depression markers, and cognitive functions. Increased socialization associated with a supervised HIIT program could favor better results concerning these outcomes. Finally, we evaluated the acceptability of the two exercise programs. We hypothesized that introducing HIIT into a rehabilitation program would be similar to, or slightly more effective than, MICT alone for improving CRF and other modifiable cardiovascular risk factors owing to a higher cardiovascular stimulation.

## Materials and methods

### Design

A 1-year follow-up randomized control trial was performed with participants randomly allocated to one of the three groups: HIIT + MICT (a combined program that included HIIT and MICT), MICT (a standard exercise program), and control (usual care with no additional exercise program). This trial was registered (27). The allocation ratio was 1:1:1, and randomization was performed by a web-based randomization system following the baseline evaluation: age, sex, and diagnosis (stroke or TIA) were controlled in the randomization. This study was approved by the institutional ethics committees of both the University du Québec à Trois-Rivières and the Centre Intégré Universitaire de Santé et de Services Sociaux de la Mauricie-et-du-Centre-du-Québec (CIUSSS-MCQ) University Hospital (CER-17-241-10.04 and CÉR-2017-002). Procedures were followed according to the Helsinki Declaration of the World Medical Association. All participants provided their written consent before taking part in the study. A pilot study that aimed to assess the feasibility and acceptability of HIIT + MICT in this population was conducted previously and the results were used to design the methodology of this study.

### Participants

Participants were recruited between January and July 2018 from medical clinics in Trois-Rivières, Québec, Canada, and from responses to invitations published in the local newspapers. Inclusion criteria were ischemic stroke or TIA with a minimum of 3 months post event and no maximum; age 40 years and over; ambulatory capacity over 10 min without or with assistive devices as needed and not currently participating in formal rehabilitation. Exclusion criteria were: TIA with isolated sensory symptoms, visual changes, or vertigo; presence of brain hemorrhage, vascular malformations, tumor, abscess; diagnostic of cognitive impairment limiting task comprehension; musculoskeletal disorders that could prevent physical activity practice; lower extremity claudication and all absolute contraindications to exercise testing according to the American College of Sports Medicine (28).

Baseline data included medical data on stroke, TIA, medication, and comorbidities that were collected by consulting the medical file in the hospital's archives with the participant's agreement before the evaluation. Baseline data including socio-demographic characteristics were collected at the first evaluation by questioning the participants directly.

### Outcome measures

The primary outcome involved potential changes in CRF determined with estimated peak oxygen uptake (VO_2_ peak) at the university hospital. Secondary outcomes were resting systolic and diastolic blood pressures, lipid profile, HbA1c, waist circumference, body composition, self-reported physical activity, functional level, anxiety and depression, and cognitive functions. These tests were performed at baseline (T0), at the end of the exercise program (T6), and 6 months later (T12) at the university kinesiology clinic or hospital. Participants' acceptability of the exercise programs was assessed at T12.

Before the exercise program, each participant underwent a clinical examination to evaluate cardiovascular risk factors and a symptom-limited graded exercise test (GXT) (see below) to determine CRF, peak power output (PPO) and the intensity of exercise during the exercise program.

#### Estimated VO_2_ peak

The GXT protocol was performed on a semi-recumbent ergocycle with 12-lead ECG monitoring (MAC 5500HD, GE Healthcare, USA). The cadence was maintained at 60 rpm; power started between 0 and 60 watts depending on the estimated participant's capacity and increased progressively by 10 watts per minute. Blood pressure response was assessed manually with a sphygmomanometer (Hillrom, Welch Allyn Tycos, USA) every 3 min. Systolic blood pressure > 250 mm Hg or diastolic blood pressure > 115 mm Hg were considered an absolute indication for terminating the GXT (12). According to ACSM guidelines (28), test termination criteria also included volitional fatigue, significant arrhythmia, evidence of ischemia, angina-like symptoms, a drop in systolic blood pressure of ≥10 mm Hg despite an increase in work rate or below the value obtained on the ergocycle prior to testing, shortness of breath, wheezing or leg cramps, signs of poor perfusion, failure of heart rate to increase with increased intensity or the participant's request to stop (28). The majority of GXT (95%) were stopped because of volitional fatigue, and other reasons were a patient's request to stop because of pain in the leg (4%) and a decrease of systolic blood pressure below the prior test value (1%). PPO was established as the highest power maintained during 60 s during the final stage of the GXT and was used to determine exercise intensity during the exercise program. This value was used to estimate VO2 peak using the ACSM equation (28). The GXT protocol was also assessed at 3 months (T3) in the two exercise groups to adjust exercise intensity.

Resting systolic and diastolic blood pressures were measured twice on each arm with the participant in a sitting position. The result was the average of the two measures taken with an automated sphygmomanometer (HEM-907XL, Omron IntelliSense, USA) in accordance with the recommendations of the Canadian Education Hypertension Program (29). The resting heart rate was simultaneously recorded, and the average was reported. After overnight fasting, blood samples were collected in the morning, centrifuged and stored as serum at −80°C until analysis. Low-density lipoprotein (LDL), high-density lipoprotein (HDL), triglycerides, total cholesterol, and HbA1c were analyzed with standardized procedures at the Centre Hospitalier Universitaire Régional de Trois-Rivières, Québec, Canada (Advia XPT, Siemens, Erlangen, Germany). Waist circumference was measured in a standing and relaxed position using a flexible measuring tape. Body weight and height were measured with a stadiometer (402LB, Health-o-meter, USA) and were used to calculate body mass index (BMI). Body fat mass was calculated with bioelectrical impedance (BC-418, TANITA, USA). Self-reported physical activity was recorded with the Godin Leisure-Time Exercise Questionnaire (GLTEQ) (30). The Short Physical Performance Battery (SPPB) was used as an indicator of functionality and frailty (31). The Hospital Anxiety and Depression Scale (HAD) was used to evaluate psychological distress (32). The Montreal Cognitive Assessment (MoCA) was used to evaluate cognitive function. The test has good sensitivity and specificity for detecting mild cognitive impairment in stroke or TIA patients (33). The acceptability of the intervention was assessed with a French version of the Treatment Acceptability and Preferences Questionnaire (TAPQ) (19) for the two exercise groups. The questionnaire was administered at T12 by an independent evaluator. The TAPQ evaluates acceptability based on four items: effectiveness, suitability, appropriateness, and willingness. All were answered on a 5-point scale ranging from “not at all” (0) to “extremely” (4).

### Exercise programs

Both exercise programs lasted 6 months, and both included three weekly aerobic sessions. Supervised exercise sessions were performed at the university kinesiology clinic while unsupervised sessions took place at home. Each supervised session was conducted on an upright ergocycle (Ergomedic 828E, Monark, Sweden). This exercise mode was chosen because it is less limiting for patients with gait and balance impairment or muscle weakness related to hemiparesis. Symptoms or evidence of exercise intolerance were checked during exercise under clinical supervision administered by a qualified kinesiologist. Heart rate was recorded continuously with a Polar FT4 monitor (Polar Electro, Finland). Blood pressure was measured on the right arm manually with a sphygmomanometer (Hillrom, Welch Allyn Tycos, USA) and a stethoscope (Littmann, Canada) before and after exercise following 5 min of rest in a sitting position according to the recommendations of the Canadian Education Hypertension Program (29). During exercise, blood pressure was measured every 5 min with the same material. The maximal limit was set at 220 mm Hg systolic and 110 mm Hg diastolic. A drop of 20 mm Hg greater or lesser than the resting level was also considered a hypotensive response requiring exercise cessation (28). Perceived exertion was questioned during exercise and was rated at the end of each exercise session based on a 10-level perceived exertion scale and the average heart rate.

#### MICT group

The intervention consisted of an exercise program involving three weekly aerobic sessions including a combination of one MICT session performed under clinical supervision at a university kinesiology clinic and two MICT sessions performed at home. Supervised exercise was done on an upright ergocycle (Ergomedic 828E, Monark, Sweden) at 50% of PPO. Each session included a 5-min warm-up and a 5-min cool-down at 40% of PPO. Exercise time progressed from 20 to 40 min over the intervention and was adjusted according to the participant's tolerance. The two other MICT sessions were done at home and included 30 min of aerobic exercise at moderate intensity determined by the participant's perceived exertion (target: 4–6/10). Participants could choose their preferred exercise (e.g., walking, swimming, dancing, or cycling) and divide their sessions into 10-min minimum bouts as needed. Weekly self-reported home exercises were noted in participants' files during the supervised session by the clinician.

#### HIIT + MICT combined group

**Figure 1:**
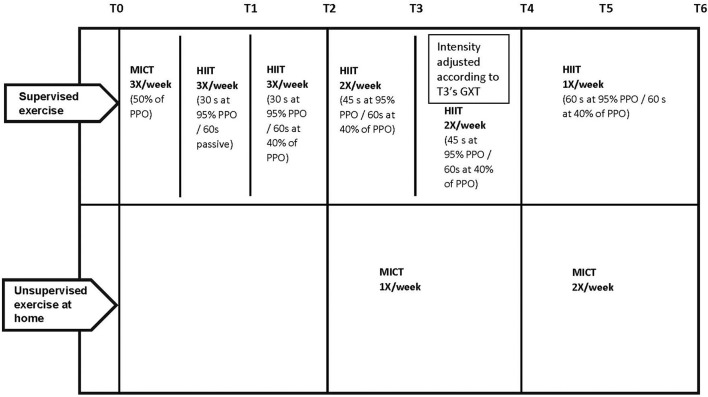
Timeline for HIIT + MICT group. PPO, peak power output; GLTQ, godin leisure time questionnaire; HAD, hospital anxiety and depression scale; MoCA, montreal cognitive assessment; SPPB, short physical performance battery; HIIT, high intensity interval training; MICT, moderate intensity continuous training; T0, baseline; T3, 3-months; T6, 6-months; T12, 12-months.

The intervention involved an exercise program consisting of three weekly aerobic sessions including a combination of progressive low-volume HIIT sessions performed under clinical supervision at the university kinesiology clinic and MICT sessions mostly performed at home. A combined program was chosen to create a realistic exercise program that could be implemented in a clinical setting and allow patients to exercise without clinical supervision as well. The rationale behind this program is that, since HIIT must be conducted under clinical supervision with high-risk patients (11), it cannot be prescribed at home. As MICT is easily transferable to the home, it represents a key factor that promotes the patient's perseverance in a long-term perspective of physical activity adhesion. Thus, the combination of both supervised HIIT and unsupervised MICT could provide the physiological benefits of HIIT while allowing patients to develop long-term adhesion to physical activity (52). Therefore, according to this objective, the program was designed to decrease supervision over time while continuing to maintain a frequency of three times by week. This reduced supervision is shown in Figure 1. During the first 2 months, patients were required to perform three supervised HIIT sessions weekly. During the next 2 months, they had to perform two supervised HIIT sessions and one unsupervised MICT session weekly. The two last months were composed of one supervised HIIT session and two unsupervised MICT sessions. To initiate physical activity and gradually introduce HIIT during the first week, participants began with three supervised MICT. In the second week, HIIT was introduced, but initially with passive recovery, and active recovery was added after 1 month. A total of 44 HIIT sessions and 44 MICT sessions per participant were expected. HIIT was performed on an upright ergocycle (Ergomedic 828E, Monark, Sweden) and included several bouts at 95% of peak power output (PPO) interspersed with a 60-s recovery. Time at 95% of PPO progressed from 30- to 60-s as shown in Figure 1. Recovery was passive in the first month and progressed to active at 40% of PPO for the remainder of the intervention. Each session included a 5-min warm-up and a 5-min cool-down at 40% of PPO. Exercise time progressed from 20 to 40 min over the intervention and was adjusted according to the participant's tolerance and the progress of the protocol. As participants did a GXT after 3 months, resistance was adjusted based on the new PPO obtained. Participants were asked to perform their MICT sessions at home. MICT included 30 min of aerobic exercise at moderate intensity determined by participants' perceived exertion (target: 4–6/10). Participants could choose their preferred exercise (e.g., walking, swimming, dancing, or cycling) and divide their sessions into 10-min minimum bouts as needed. Weekly self-reported home exercises were noted in participants' files during the supervised session by the clinician.

### Control group

The control group received the usual care without any additional physical activity counseling or interaction with study personnel between evaluations other than the routine recommendations from their family physicians.

### Statistical analysis

The equivalence of baseline participants' characteristics was assessed with analyses of variance (ANOVA) by comparing the three groups for linear data or Chi-square tests for discrete data. The repeated measure ANOVA (Group × Time) was performed to compare outcome variables between the three groups (HIIT + MICT, MICT, and control) across time points (T0, T6, and T12). Because of certain variables' lack of conformity to theoretical statistical assumptions, especially heteroscedasticity and positive distribution skewness indices, we resorted to the Monte-Carlo simulation method for estimating the F probabilities (based on 1,000,000 iterations). Pearson's *r* coefficient was used to measure the correlation between changes in self-reported physical activity and change in VO_2_peak. Descriptive statistics served to summarize baseline characteristics and acceptability results. Statistical significance was set at *p* < 0.05 and data are expressed as mean and standard deviations (SD). Analyses were conducted using Microsoft Excel 2010 and IBM SPSS Statistics (version 26).

Based on a priori power analysis, a sample size of 42 participants at 12-month follow-up (14 participants × 3 groups) was deemed adequate for the repeated measures ANOVA with an expected large effect size between exercise groups vs. control group (*f*^2^ > 0,8), type I error rate of 5% and power of 80%. The large effect size was estimated based on the literature regarding the effect of a MICT program on CRF. Based on our previous pilot study, we anticipated a 30% dropout rate; hence, a total of 60 participants were expected at baseline.

## Results

**Figure 2:**
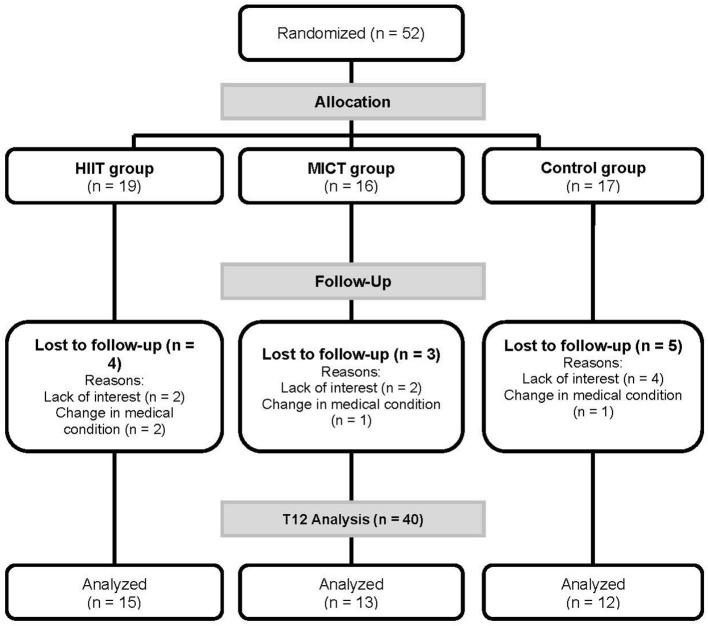
Flow chart. HIIT, high intensity interval training; MICT, moderate intensity continuous training.

The baseline sample was composed of 52 participants (33 men and 19 women). Participants' age was 69.2 ± 10.7 years; 39 had suffered a stroke and 13 a TIA, of whom six had multiple strokes or TIA. All participants were living in the community and none participated actively in stroke rehabilitation. The baseline characteristics are shown in Table 1. The medication used was similar between groups, whereas the use of an orthopedic device and the presence of atrial fibrillation differed from group to group. As described in Figure 2, 52 participants underwent the initial evaluation and were randomized to an HIIT + MICT group (*n* = 19), a MICT group (*n* = 16), and a control group (*n* = 17). The attrition rate was 23% at T12. Indeed, thirteen participants were lost to follow-up and did not complete the 12-month evaluation. Their characteristics, however, were shown to be similar to those who completed the protocol. The main reasons for dropout were lack of interest, especially in the control group (*n* = 8), and a change in health status that limited participation in the exercise program (*n* = 4). Thus, 40 participants (77%) completed both baseline and the 12-month evaluation. No adverse events were registered during the exercise programs. A total of 802 HIIT sessions were performed by those who remained in the HIIT + MICT group. Exercise attendance, defined as the mean percentage of total supervised exercise sessions completed, was 95% (79–100%) for the HIIT+MICT group and 93% (77–100%) for the MICT group. Mean self-reported home exercise attendance was 78 ± 23% (17–100%) for the HIIT + MICT group and 82 ± 18% (42–100%) for the MICT group. The exercise attendance (supervised and non-supervised) was not different between HIIT + MICT and MICT groups (*p* > 0.05). Although the use of an orthopedic device and the presence of atrial fibrillation differed from group to group, these characteristics had no impact on baseline VO_2_peak (*p* > 0.05). At baseline, a higher VO_2_peak was associated with male sex, younger age, and no multiple stroke or TIA (Pearson correlation: all *p* < 0.05). However, these characteristics had no impact on the change in VO_2_peak following the intervention (Pearson correlation: all *p* > 0.05). Thus, all our baseline characteristics did not have a significant impact on the change in VO_2_peak.

**Table 1:** Baseline characteristics of the participants.

		**HIIT + MICT**	**MICT**	**Control**	**Total**	***P*-value**
		**(*N* = 19)**	**(*N* = 16)**	**(*N* = 17)**	**(*N* = 52)**	
**Socio-demographic characteristics**						
Sex—*n* (%)	Male	13 (68.4)	10 (62.5)	10 (58.8)	33 (63.5)	NS
	Female	6 (31.6)	6 (37.5)	7 (41.2)	19 (36.5)	
Age—years		71.8 ± 9.9	65.6 ± 11.3	69.6 ± 10.7	69.2 ± 10.7	NS
Employment status	Employed	5 (26.3)	4 (25.0)	3 (17.6)	12 (23.1)	NS
	Retired	14 (73.7)	12 (75.0)	14 (82.4)	40 (76.9)	
Marital status	Cohabitation	13 (68.4)	13 (81.3)	10 (58.8)	36 (69.2)	NS
	Single	6 (31.9)	3 (18.8)	7 (41.2)	16 (30.8)	
**Health characteristics**						
Neurovascular disease—*n* (%)	Stroke	11 (57.9)	14 (87.5)	14 (82.4)	39 (75.0)	NS
	TIA	8 (42.1)	2 (12.5)	3 (17.6)	13 (25.0)	
Time since the event—months		37.3 ± 61.6	51.8 ± 78.7	29.3 ± 39.1	39.2 ± 61.0	NS
Multiple stroke or TIA—*n* (%)		3 (15.8)	1 (6.3)	2 (11.8)	6 (11.5)	NS
VO_2_peak (ml·min^−1^·kg^−1^)		19.5 ± 5.3	20.4 ± 4.7	19.3 ± 7.3	19.7 ± 5.8	NS
Use of orthotic device—*n* (%)		1 (5.3)	5 (31.3)	1 (6.0)	7 (13.5)	0.043
BMI—kg/m^2^		27.9 ± 2.4	28.4 ± 5.4	28.3 ± 5.9	28.2 ± 4.7	NS
BF—%		30.2 ± 5.0	31.1 ± 6.5	32.1 ± 8.4	31.1 ± 6.6	NS
Smoking habits	Non-smoker	6 (31.9)	4 (25.0)	6 (35.3)	16 (30.8)	NS
	Smoker	0 (0)	1 (6.3)	3 (17.6)	4 (7.7)	NS
	Ex-smoker	13 (68.4)	11 (68.8)	8 (47.1)	32 (61.5)	NS
Hypercholesterolemia—*n* (%)		15 (78.9)	13 (81.3)	14 (82.4)	42 (80.8)	NS
Diabetes—*n* (%)		4 (21.1)	4 (25.0)	4 (23.5)	12 (23.1)	NS
Hypertension—*n* (%)		10 (52.6)	11 (68.8)	11 (64.7)	32 (61.5)	NS
PAD—*n* (%)		1 (5.3)	3 (18.8)	0 (0)	4 (7.7)	NS
CHD—*n* (%)		6 (31.6)	4 (25.0)	1 (6.0)	11 (21.2)	NS
CKD—*n* (%)		3 (15.8)	1 (6.3)	2 (11.8)	6 (11.5)	NS
AF—*n* (%)		6 (31.6)	0 (0)	2 (11.8)	8 (15.4)	0.032
**Medication**						
Lipids lowering drugs		16 (84.2)	11 (68.8)	13 (76.5)	40 (76.9)	NS
Antihypertensives drugs		12 (63.2)	10 (62.5)	12 (70.6)	34 (65.4)	NS
Anticoagulants		8 (42.1)	4 (25.0)	4 (23.5)	16 (30.8)	NS
Antiplatelets		14 (73.7)	14 (87.5)	15 (88.2)	43 (82.7)	NS
Hypoglycemic drugs		2 (10.5)	1 (6.3)	4 (23.5)	7 (13.5)	NS
Anxiolytics		3 (15.8)	3 (18.8)	1 (5.9)	7 (13.5)	NS
Antidepressants		7 (36.8)	4 (25.0)	1 (5.9)	12 (23.1)	NS

Data are presented as mean ± SD or *n* (%). TIA, transient ischemic attack; BMI, body mass index; BF, body fat; PAD, peripheral arterial disease; CHD, coronary heart disease; CKD, chronic kidney disease; AF, atrial fibrillation. *P*-value represents the comparison between groups; NS, non-significant.

**Figure 3:**
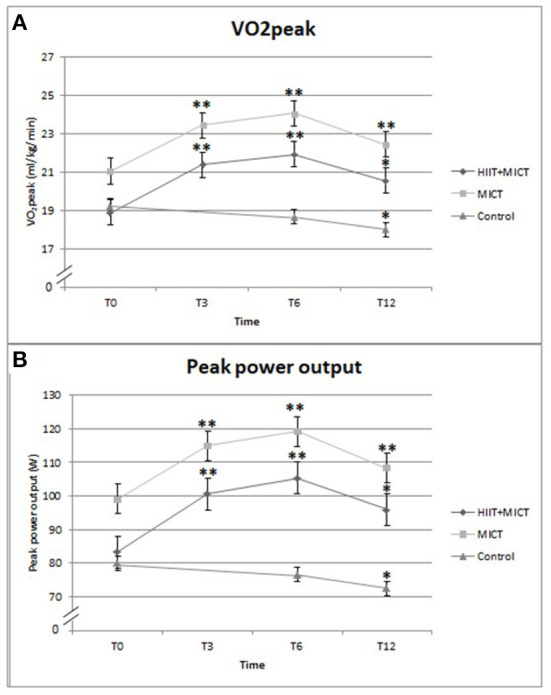
Comparison the effects of exercises programs on **(A)** VO_2_peak, **(B)** peak power output. *Significantly different compared to T0 by *p* < 0.05, **significantly different compared to T0 by *p* < 0.01.

The potential impacts of the two exercise interventions on VO_2_peak and PPO are shown in Figure 3 and quantified in Table 2 (results for the 39 participants who completed the 12-months follow-up for these measurements). For both variables, a strong Group × Time interaction effect emerges (*F* = 9.222 for VO_2_peak and 10.522 for PPO, both with: df_1_ = 4, df_2_ = 72; *p* < 0.001). As seen in Figure 3, both measures increase sharply from T0 to T6 and then decrease somewhat at the recall measurement. In the control group, the corresponding values decrease slightly (*p* < 0.027 for VO_2_peak and *p* < 0.020 for PPO, tested as a linear trend with *F*_1, 72_). A finer analysis using an orthogonal decomposition of the interaction effect shows that 99.6% (VO_2_peak) and 98.5% of the total Group × Time variance are due to the contrast between the (merged) exercise groups and the control group, leaving a quasi-null interaction variance between the exercise groups themselves.

**Table 2:** Change in primary and secondary outcomes.

		**T0**	**T6**	**T12**	**Repeated-measure ANOVA**		
					***p*** **-values**		
					**Group**	**Time**	**Interaction**
**Exercise and physical variables**							
VO_2_peak (ml·min^−1^·kg^−1^) (*n* = 39)	HIIT + MICT	18.9 ± 5.5	21.9 ± 6.1	20.6 ± 5.6	0.297	< 0.001^*^	< 0.001^*^
	MICT	21.1 ± 4.5	24.1 ± 4.9	22.5 ± 5.3			
	Control	19.3 ± 8.4	18.7 ± 8.0	18.0 ± 7.6			
PPO (W) (*n* = 39)	HIIT + MICT	83.3 ± 41.9	105.3 ± 48.4	96.0 ± 44.1	0.166	< 0.001^*^	< 0.001^*^
	MICT	99.2 ± 35.5	119.2 ± 35.0	108.3 ± 37.4			
	Control	80 ± 49.4	76.7 ± 46.2	72.5 ± 45.4			
SBP (mmHg)	HIIT + MICT	125.3 ± 13.9	121 ± 17.2	123.4 ± 12.7	0.123	0.088	0.173
	MICT	130.3 ± 17.0	136.9 ± 19.8	128.9 ± 12.8			
	Control	132.3 ± 18.7	138.8 ± 14.5	127.4 ± 16.0			
DBP (mmHg)	HIIT + MICT	65 ± 12.3	66 ± 14.5	65.3 ± 7.8	0.243	0.264	0.454
	MICT	68.0 ± 9.5	73.9 ± 10.5	67.2 ± 12			
	Control	70.7 ± 9.6	73.7 ± 8.9	73.3 ± 10			
HR (beats/min)	HIIT + MICT	69.3 ± 11.3	67.4 ± 11	69.5 ± 10	0.985	0.474	0.785
	MICT	68.7 ± 13.6	68 ± 11.5	68.7 ± 11.3			
	Control	71.4 ± 11.3	68.6 ± 13.4	67.5 ± 13.2			
SPPB	HIIT + MICT	9.4 ± 3.2	10.4 ± 2.8	10.3 ± 2.3	0.373	0.458	0.003^*^
	MICT	9.3 ± 2.3	9.9 ± 2.8	10.3 ± 2.6			
	Control	9.4 ± 2.3	8.8 ± 2.5	8.1 ± 2.5			
**Anthropometric variables**							
BMI (kg/m^2^)	HIIT + MICT	28 ± 2.6	28.3 ± 2.9	28.7 ± 2.7	0.987	0.269	0.065
	MICT	28.3 ± 5.5	28.2 ± 5.3	28.4 ± 4.3			
	Control	28.5 ± 6.6	28.9 ± 6.6	28.4 ± 6			
WC (cm)	HIIT + MICT	99.3 ± 8.8	98.9 ± 9.8	99.5 ± 9.6	0.900	0.549	0.641
	MICT	97.2 ± 12.9	98.0 ± 12	98.4 ± 12.2			
	Control	99.7 ± 16.2	100.8 ± 15.8	99.9 ± 16.1			
FM (%)	HIIT + MICT	30.9 ± 5.5	32.6 ± 4.8	32.8 ± 6.0	0.736	0.227	0.514
	MICT	30.8 ± 6.9	30.4 ± 8.1	30.7 ± 7.5			
	Control	32.5 ± 9.8	33 ± 9.4	33.2 ± 11			
**Blood variables**							
HDL (mmol/L)	HIIT + MICT	1.4 ± 0.6	1.5 ± 0.6	1.4 ± 0.5	0.946	0.001^*^	0.159
	MICT	1.3 ± 0.4	1.4 ± 0.5	1.4 ± 0.4			
	Control	1.3 ± 0.3	1.4 ± 0.3	1.3 ± 0.3			
LDL (mmol/L)	HIIT + MICT	1.7 ± 0.5	1.9 ± 0.6	1.9 ± 0.7	0.264	0.003^*^	0.397
	MICT	1.6 ± 0.5	1.7 ± 0.4	1.8 ± 0.5			
	Control	1.7 ± 0.7	2.2 ± 0.9	2.2 ± 0.5			
					***p*** **-values**		
					**Group**	**Time**	**Interaction**
HbA1c	HIIT + MICT	0.061 ± 0.008	0.061 ± 0.012	0.061 ± 0.010	0.603	0.272	0.903
	MICT	0.057 ± 0.004	0.059 ± 0.008	0.058 ± 0.005			
	Control	0.059 ± 0.007	0.061 ± 0.008	0.061 ± 0.009			
**Questionnaires**							
GLTQ	HIIT + MICT	15.5 ± 13.5	34.3 ± 20.1	29.5 ± 22.2	0.002^*^	0.005^*^	0.016^*^
	MICT	27.5 ± 25.1	46.4 ± 19.4	38.2 ± 24.1			
	Control	18 ± 13.6	12.6 ± 11.4	14.8 ± 12.0			
HAD	HIIT + MICT	11.1 ± 4.1	7.6 ± 3.6	7.4 ± 3	0.256	0.001^*^	0.014^*^
	MICT	11.8 ± 7.2	9.2 ± 4.4	9.3 ± 6.2			
	Control	11.3 ± 6.1	11.6 ± 4.4	12.0 ± 4.2			
MoCA	HIIT + MICT	23.9 ± 3.9	24.7 ± 3.4	25.6 ± 3.3	0.209	0.056	0.041^*^
	MICT	23.6 ± 2.5	24.1 ± 3.5	25 ± 3.4			
	Control	22.9 ± 4.1	21.9 ± 4.8	22.2 ± 5.3			

Data are presented as mean ± SD; PPO, peak power output; GLTQ, Godin leisure time questionnaire; HAD, hospital anxiety and depression scale; MoCA, montreal cognitive assessment; HDL, high density lipoprotein; LDL, low density lipoprotein; SBP, systolic blood pressure; DBP, diastolic blood pressure; HR, heart rate; BMI, body mass index; WC, waist circumference; FM, fat mass; SPPB, short physical performance battery. ^*^Results statically significant (*p*-value < 0.05).

As shown in Table 2, self-reported physical activity assessed by the GLTQ score presented a pattern similar to VO_2_peak and PPO with a Group × Time interaction effect (*p* = 0.016). The GLTQ score increased similarly for the HIIT + MICT and MICT groups after the 6-month exercise program, but decreased for the control group. There was a positive correlation between the change in VO_2_peak and the change in GLTQ after the 6-month of exercise program (*r* = 0.5; *p* < 0.01).

All other secondary outcome measures are presented in Table 2. As shown, the HIIT + MICT and MICT groups presented a decreased Hospital Anxiety and Depression Scale score compared to the control group that remained unchanged, as reflected by a significant interaction effect Group × Time (*p* = 0.014). The functional level described by the Short Physical Performance Battery score had evolved in different ways for exercise groups and control group with a significant interaction effect Group × Time (*p* = 0.003), but without a significant global time effect (*p* = 0.458). A Group × Time interaction effect (*p* = 0.041) was also present for the cognitive functions explained by a different evolution of the MoCA score between control and exercise groups without significant time effect (*p* = 0.056). Cardiovascular risk factors did not change significantly over the intervention time.

All acceptability attributes were evaluated with the TAPQ ranging from 3/4 to 4/4 for the HIIT+MICT and MICT groups (3.6 for effectiveness; suitability 3.7; appropriateness 3.5, and willingness 3.8) with no difference between groups (Table 3).

**Table 3:** Acceptability of HIIT + MICT and MICT program assessed by TAPQ.

**Acceptability attributes (score 0–4)**	**HIIT + MICT**	**MICT**	***P*-values**
Effectiveness	3.6 [3, 4]	3.4 [2–4]	NS
Suitability	3.6 [3, 4]	3.8 [3,4]	NS
Appropriateness	3.5 [2–4]	3.4 [1–4]	NS
Willingness	3.7 [3, 4]	3.8 [3, 4]	NS

Data are presented as mean (range). *P*-value represents the comparison between groups. NS, non-significant.

## Discussion

This randomized control trial demonstrated that a 6-month combined HIIT and MICT program or a standard MICT exercise program induced a similar improvement in PPO and estimated VO_2_peak among patients with ischemic stroke or TIA compared to a control group. These effects seem to persist 6 months after the end of the programs despite a slight decrease in estimated VO_2_peak and PPO at T12 compared to T6. Both exercise programs also induced a similar increase in self-reported physical activity as well as a decrease in anxiety and depression markers. Participants gave both the HIIT + MICT and MICT programs a very good acceptability evaluation. Our results do not support any superiority of HIIT + MICT over a standard exercise program using MICT only. No adverse events occurred during the two exercise programs, which corroborates the available literature suggesting that HIIT is safe for stroke survivors.

Considering that activities of daily living generally require between 3 and 5 METs [4] and that our population reached on average 5.6 METs of VO_2_peak, many daily living activities are unsustainable. Shephard suggests that a VO_2_peak level below 15 and 18 ml·kg^−1^·min^−1^ for women and men, respectively, leads to a loss of independence in elderly people (34). Furthermore, it is important to consider that the energy cost of walking is 1.5 times higher in stroke patients than in matched controls (35). Even light ambulatory activities may require a moderate to high level of effort and exercise can prevent functional independence (36). Therefore, whether the small improvement of 3 ml·kg^−1^·min^−1^ induced by our two exercise programs impacts functionality and quality of life remains to be demonstrated. In our study, the functionality score assessed by SPPB did not increase significantly after the exercise programs, but it can be explained by the fact that many participants reached the maximal score even at baseline. However, the significant interaction effect demonstrated that the exercise groups and control group evolved in different ways over time. Moreover, because physical inactivity is highly prevalent after a stroke (12), a small dose of exercise could have a significant impact on CRF (9). The literature indicates that an improvement of only 1 MET is associated with a 10–25% improvement in survival (37).

To our knowledge, there is no comparable randomized trial regarding the effect of a combined MICT-HIIT program on stroke patients. In our study, the increased VO_2_peak following the intervention represents an improvement of 16.8 and 15.2% for HIIT + MICT and MICT, respectively. These results are comparable to the mean gain of 12.5% reported by Billinger et al. (9) in a review of 14 studies where exercise intensity was generally moderate. In a pilot study on chronic stroke, Gjellesvik et al. (38) reported a VO_2_peak increase of 11.6% after only 4 weeks of HIIT. Most studies of HIIT in stroke focused on treadmill walking given the task specificity to improve gait and functional outcomes (15). However, we decided to use the ergocycle in order to include a larger population, not only individuals able to walk without physical assistance. Regarding the HIIT protocol, our choice was based on suggestions made by Boyne et al. (39). After within-session comparison of three different HIIT protocols, these authors suggested beginning with a combination of 30-s bursts at maximal tolerated speed (on treadmill) intercepted with 60-s recovery to optimize aerobic intensity. To progress our protocol, we chose to increase burst to 45- and 60-s over time while maintaining 60-s recovery. Then, the last months of HIIT were conducted with a 1:1 ratio, which also conforms to the suggestions of Boyne et al. (39). While some authors demonstrate that higher intensities of exercise are most effective for improving CRF (40), our results do not suggest the superiority of adding HIIT in stroke or TIA patients. The fact that our HIIT + MICT program was a combination of HIIT and MICT can explain the lack of difference because the stimulus was not very different and the purpose was not to directly compare MICT and HIIT. Thus, consequent to our results, we cannot conclude that a combination of HIIT and MICT offers any benefits compared with a standard physical activity program using MICT alone. In the same vein, a preliminary review of Wiener et al. including six studies and 140 participants showed that HIIT may be an effective rehabilitation intervention but is not superior to MICT in terms of CRF improvement (41). Similarly, Ellingsen et al., did not report superiority of HIIT compared with MICT in patients with heart failure with reduced ejection fraction (42). HIIT has been studied in clinical populations with hypertension, heart failure, type 2 diabetes and metabolic syndrome and, in these settings, health benefits seem to be similar or superior to MICT despite a shorter duration. In our study, exercise time was similar between both exercise groups without the superiority of HIIT + MICT. Based on our results and those of other researchers, the challenge is to initiate an exercise program for patients having the most sedentary lifestyle despite physical activity recommendations. Blennerhasset et al. (43) recently reported that among stroke patients, the most common barrier is the difficulty of starting an exercise program. Accordingly, we believe it's essential to discover ways to have stroke patients practice exercise regularly, regardless of type of exercise. MICT or HIIT + MICT could both be effective options depending on patient preference. Moreover, our acceptability results demonstrate that participants appreciated both the HIIT + MICT and MICT programs.

Another important challenge is to maintain long-term engagement in regular physical activity in order to sustain the benefits. Our two exercise programs had the same impact on self-reported physical activity until 6 months after the end of the program (T12). Despite a slight decrease compared with T6 (probably related to the decrease of exercise level reported), the HIIT + MICT and MICT groups showed significant VO_2_peak improvement compared with baseline (T0) and a higher level of self-reported physical activity compared with the control group. Gunnes et al. (44) demonstrated that stroke patients who receive individualized monthly coaching maintain moderate-to-good adherence to long-term daily physical activity. We chose to design our HIIT + MICT program combining supervised HIIT (for physiological benefits) and unsupervised MICT (easily done at home) to create a realistic program that patients can maintain over time, and which would gradually result in fewer clinical sessions to help patients maintain their physical activity practice. Interestingly, despite the fact that our HIIT + MICT group had twice as many supervised sessions as the MICT group, our results showed a similar improvement in CRF, self-reported physical activity and anxiety and depression markers. This leads us to believe that even though stroke patients need clinical support to start an exercise program, a weekly follow-up could offer sufficient benefits. However, individualization is still necessary, and some patients may need more supervision as shown by our self-reported home exercise attendance ranging from 17 to 100%. In a long-term perspective, it is important to set up exercise programs that promote the patient's perseverance in physical activity practice. It was recently suggested that without supervision, individuals assigned to a HIIT program exercise at lower intensities than expected, and HIIT groups show no more long-term adherence than MICT (52). In another intervention regarding cardiac rehabilitation, the lack of VO_2_peak improvement was ascribed to “…lack of adherence to the prescribed home-based HIIT program…” (53). Our study suggests that patients could choose one or the other program (HIIT + MICT or MICT alone) and derive benefits from both.

The literature is unclear regarding CRF improvement following exercise in stroke patients and its potential effect on cardiovascular risk factors. Some authors observed favorable changes in systolic blood pressure (45), insulin resistance and glucose tolerance (46), total cholesterol and triglycerides (47), but these improvements were slight and inconsistent. Most studies focused on MICT and low intensity exercise, but even with the inclusion of higher intensity exercise, our results did not support improvement regarding these factors. Note, however, that at baseline, the mean values of the cardiovascular risk factors were in the therapeutic targets for all our groups according to the Canadian Cardiovascular Society. This, therefore, could explains the lack of change in these factors in our groups as well as the absence of interventions related to nutrition.

Anxiety and depression are, for the most part, significantly higher in stroke patients than in the general population (22). They can negatively affect recovery and seem to persist many years after the event (48). This problem therefore represents a priority intervention in the management of stroke patients. In view of the positive results obtained by those in the HIIT + MICT and MICT groups on the HAD score, we believe that physical activity can effectively reduce anxiety and depression markers among these patients. These results are consistent with those of studies showing lower levels of anxiety and depression among the physically active elderly (24) and with authors who suggest that exercise can be a form of treatment for depression (49). However, those improvements can also be influenced by the social interaction provided by the intervention, not only the effect of exercise itself.

Regarding cognitive functions, the literature suggests that an increase in physical activity, especially aerobic exercise, can improve executive functions and promote neuroplasticity through an increase in brain blood flow (50). However, these potential benefits of exercise are not clearly demonstrated after stroke (26), a fact explained by the small number of studies with heterogeneous interventions. According to Crozier et al., we can extrapolate the potential contribution of HIIT on neuroplasticity post-stroke based on results from other populations; they also suggest that intensity is the key factor to enhance the expression of neurotrophins that increase neural repair processes (15). Our results cannot support an effect of exercise on cognitive functions even with inclusion of high intensity exercise. The interaction effect created by the different evolution of the MOCA score between exercise groups and control group and the time effect showing a trend (*p* = 0.056) leads us to believe that a larger sample size could produce positive results. Larger studies are needed to confirm this.

Regarding safety, a supervised HIIT program was used instead of a home program in order to receive ethical approval. Our results and those of other studies, however, suggest that home-based HIIT is as safe as MICT (55). We also believe that home-based HIIT is more difficult to pursue for non-athletic participants, as recently shown by Ekkekakis and Biddle (52).

### Limitations and strengths of the study

Certain limitations in this study need to be addressed. First, the study sample was limited to voluntary participants, possibly leading to selection bias and potential bias by the exclusion of dropouts in the acceptability study. This may reflect their greater adherence to exercise compared with patients who declined to participate. Also, for certain outcomes, more participants would have increased statistical power. Second, because the unsupervised at-home exercises were guided by participants' perceived exertion and were self-reported, it is difficult to quantify physical activity performed at home. Moreover, attendance for home exercise presented a large variability (range from 17 to 100%) that may influence CRF and secondary outcomes. Third, the purpose of our study was not to differentiate between HIIT and MICT. Thus, the effects of MICT and HIIT are not compared, since our HIIT + MICT group participated in a combination of both types of exercise. However, this allowed us to observe the effects of a realistic combined program that could be transferable to a clinical environment. Fourth, the individual who conducted exercise interventions could not be blinded because she assisted the cardiologist in performing the GXT. The final limitation is the broad heterogeneity of our sample regarding age, time since stroke, inclusion of TIA and comorbidities. But this limitation is also a strength insofar as it reflects the reality of stroke patients and could make our results more easily transferable to clinical settings. However, we had no participants with major motor impairments. Major impairments would have required the use of a training tool other than the upright ergocycle. For example, the semi-recumbent Nustep would have been appropriate for patients with upper limb spasticity or lack of control. Additional strengths of this study are: (1) its focus on a clinically relevant problem in a major clinical population, (2) the training programs, which include some aspects of supervision and intensity adjustment, and (3) the 6-month training duration followed by a 6-month post intervention follow-up, which may explain results not yet observed in other studies.

## Conclusion

This randomized controlled trial provides evidence demonstrating that a 6-month combination of HIIT and MICT programs or a standard MICT exercise program produces a similar improvement in VO_2_peak, self-reported physical activity and anxiety and depression markers among patients with ischemic stroke or TIA, and that these effects appear to persist over time. Both programs were similar to the control group in terms of safety. Our results also suggest that HIIT could be an effective addition to standard physical activity recommendations after stroke but do not support the superiority of including HIIT + MICT compared with a standard MICT program. Furthermore, a 6-month HIIT + MICT rehabilitation program leads to similar improvements and is no more effective than MICT alone for improving CRF and secondary outcomes measures. Although exercise is very valuable, it is unfortunately underused in post-stroke care. Our high level of acceptability for both programs, however, demonstrates that patients are likely to include physical activity when they are given appropriate clinical support.

## Data availability statement

The raw data supporting the conclusions of this article will be made available by the authors, without undue reservation.

## Ethics statement

The studies involving human participants were reviewed and approved by Institutional Ethics Committees of both the University du Québec à Trois-Rivières and the Centre Intégré Universitaire de Santé et de Services Sociaux de la Mauricie-et-du-Centre-du-Québec (CIUSSS-MCQ) University Hospital (CER-17-241-10.04 and CÉR-2017-002). The patients/participants provided their written informed consent to participate in this study.

## Author contributions

TL: literature review, methodology development, study coordination, participant evaluation, monitoring and training of participants, data gathering, statistical analyzes, results interpretation, and article writing and submission. FT: development of the methodology, results interpretation, article writing and submission, and article revision. Y-TS: participant evaluation and article revision. JH: development of the methodology, participant evaluation, results interpretation, article writing and submission, and article revision. MP: participant evaluation, monitoring and training of participants, and article revision. All authors contributed to the article and approved the submitted version.
